# Introductory evidence on data management and practice systems of forensic autopsies in sudden and unnatural deaths: a scoping review

**DOI:** 10.1186/s41935-022-00293-3

**Published:** 2022-09-19

**Authors:** Salona Prahladh, Jacqueline van Wyk

**Affiliations:** 1 Department of Forensic Medicine, Inkosi Albert Luthuli Central Hospital, Durban, 4001 South Africa; 2 Discipline of Clinical and Professional Practice, College of Health Sciences, University of KwaZulu-Natal, Durban, 4001 South Africa

**Keywords:** Unnatural death, Data practice, Data management systems, Autopsy, Post-mortem examination, Forensic Medicine, Pathology

## Abstract

**Background:**

The investigation into sudden unexpected and unnatural deaths supports criminal justice, aids in litigation, and provides important information for public health including surveillance, epidemiology, and prevention programs. The use of mortality data to convey trends can inform policy development and resource allocations. Hence, data practices/management systems in Forensic Medicine are critical. This study scoped literature and described the body of knowledge on data management and practice systems in Forensic Medicine.

**Methods:**

Five steps of the methodological framework of Arksey and O’Malley guided this scoping review. A combination of keywords, Boolean terms, and Medical Subject Headings was used to search PubMed, EBSCOhost (CINAHL with full text and Health Sources), Cochrane Library, Scopus, Web of Science, Science Direct, WorldCat, and Google Scholar from the 18th to 24th of June 2020 and updated in November 2021 for peer review papers. This study included articles involving unnatural deaths, focused on data practice or data management systems, relating to Forensic Medicine, all study designs, and published in English. Screening and selection and data extraction were conducted by two reviews. Thematic analysis was conducted, and the results were reported using both quantitatively and qualitatively.

**Results:**

Of the 23,059 articles, 16 met this study’s inclusion criteria. The included articles were published between 2008 and 2019. Eight of the 16 articles were published between 2017 and 2019. Most of the included studies were conducted in the USA (5) and Australia/New Zealand (4). Only two publications were from lower- and middle-income countries (Nigeria, Mexico), and the remaining 14 were from high-income countries (Italy, Denmark, USA, Australia, and New Zealand, Japan, Switzerland, Canada). The data management systems found in this study were as follows: Virtopsy, Canadian Coroner and Medical Examiner Database, Infant Injury Database, Fatal injury surveillance system, Medical Examiners and Coroners Alert System, National Violent Deaths Reporting System, AM/PM Database, Tokyo CDISC/ODM, and National Coronial Information System.

**Conclusions:**

This study’s results revealed limited articles relating to data management and practice systems in Forensic Medicine, particularly in LMICs through literature indicating there is a prevalence of unnatural deaths in LMICs. This study, therefore, recommends research on data management and practice systems relating to forensic medicine in LMICs to inform policy decisions.

**Supplementary Information:**

The online version contains supplementary material available at 10.1186/s41935-022-00293-3.

## Background

The global burden of trauma, particularly in low- and middle-income countries places a large strain on resources, and therefore, the diagnostic value of autopsies must be reiterated (Salona Prahladh 2018). The use of autopsies remains the gold standard in assessing standards of medical care. There is a concerning decline in autopsies even though its value to the medical fraternity is acknowledged (Aase 2013; Bagher et al. 2015). Forensic Medicine and Forensic Pathology apply scientific and medical knowledge to inquests, and the autopsy is frequently regarded as the focus of the death investigation. The investigation into sudden unexpected and unnatural deaths supports criminal justice, aids in litigation, and provides important information for public health including surveillance, epidemiology, and prevention programs (Bagher et al. 2015; Tseng et al. 2018; Barbería et al. 2018; Pan et al. 2019; Soto Martinez et al. 2019). The evidence serves to inform policy not only for injury prevention and control but also to prevent suicide, violence, or substance abuse (Barbería et al. 2018; Pan et al. 2019; Rao et al. 2005; Grills et al. 2011; Prinsloo 2019; Willcox et al. 2020).

Globally, death investigations are conducted according to prevailing legislation which differs from country to country. Historically, the coroner system was formalized into law by England’s King Richard I in 1194 with the first coroners being knights (Koehler 2016). The coroner system from England was introduced in the 1600 s by American colonists to become an important part of the death investigation system in what would become the USA, but later, the role of the office was reduced to the medicolegal examination of a body and the determination of the cause and manner of death (Koehler 2016). Throughout the middle ages, the functions of the coroner included conducting inquests, attending to and inspecting the dead, and investigating suspicious deaths.

In the USA, coroners are generally public officials with minimal to no medical training. Some coroners only serve part-time capacities, and they also had other full-time employment. The medical examiner system was introduced due to public dissatisfaction, accusations of corruption, and an increased need to have highly trained personnel in the death investigation (Koehler 2016). This led to the emergence of a separate discipline of Forensic Medicine that began in the seventeenth century (Choo and Choi 2012). The first medical examiner system was introduced in Massachusetts in 1877. In 1959, the medical subspecialty of Forensic Pathology was formally certified and medical examiners were trained in Pathology. Forensic Pathology is viewed as subspecialty in Anatomical Pathology in countries such as Canada and the UK. In countries such as South Africa and Australia, you may do training solely in Forensic Pathology for a minimum of a year (usually more) with training in Anatomical Pathology. In South Africa, the medicolegal death investigation is conducted primarily in terms of the Inquests Act (Act 58 of 1959). The medicolegal autopsies are performed by medical practitioners, but due to the large annual number of unnatural deaths and the small number of qualified Forensic Pathologists in South Africa, a large number of these autopsies are performed by colleagues with limited formal training in performing autopsies (du Toit-Prinsloo and Saayman 2012).

The fundamental essence of Forensic Pathologists’ work is to investigate and report the cause of death. The importance of reporting the cause of death is reiterated and forms the basis of The Global Burden of Disease Study (Roth et al. 2018). This comprehensive worldwide observational epidemiological describes mortality and morbidity from major diseases, injuries, and risk factors to health at global, national, and regional levels. Mortality reporting systems can help to prioritize health system investments, track progress towards global development goals, and guide scientific research (Roth et al. 2018). The Global Burden of Disease study acknowledges the need for wider adoption and improvement of these systems because continuous reporting of cause-specific mortality in many countries represents a success for global health.

Information derived from autopsies has historically been paper-documented, filed, and archived. With the current age of technology, this information can be stored and managed electronically to ensure reporting that is current, relevant, and contributory to training and service delivery, policy implementation, and social interventions. The current coronavirus pandemic has accentuated the importance of wireless technology and the use of the Internet to transcend normal communications. Due to safety reasons, much work has to be conducted remotely in many business sectors including the medical sector. General practitioners conducted consultations virtually to adhere to social distancing and safety measures, and telephonic communication and telemedicine became a necessity due to the pandemic. At this current point in time, we are forced to be open-minded to integrate technology into our daily work lives.

This scoping review was conducted to map the evidence on data management and practice systems, their use, benefits, and challenges in Forensic Medicine. The information gained on the use and availability of digital technologies and their strengths and limitations to collect autopsy data can inform models to suit similar purposes in Forensic Medicine in lower- and middle-income countries.

## Methods

This study’s protocol was developed a priori and published (Prahladh and van Wyk  2020). This study used the Arksey and O’Malley framework to conduct a scoping review which includes the following: (i) the research question was identified, (ii) relevant studies were identified, (iii) eligible studies were selected, (iv) the data was charted, and (v) the results were collated and summarized (Arksey and O'Malley 2005; PRISMA 2018).

### Identifying the research question

The main research question was “In the last 10 years, what evidence on data management and practice systems and their benefits and challenges in forensic medicine exist globally? This study’s population, concept, and context were sudden/unnatural deaths, data practices, and forensic medicine (autopsies or post-mortem examinations) globally, respectively. The research sub-questions were as follows:

1. What evidence exists on data management and practice systems in forensic medicine?
2. What are the reported benefits and challenges of the data management and practice systems used in forensic medicine?

### Identifying relevant studies

A systematic search of both gray literature and published literature was done to retrieve articles relating to data practice, use, benefits, and challenges in forensic medicine internationally. A combination of keywords, Boolean terms, and Medical Subject Headings was used to search PubMed, EBSCOhost (Academic Search Complete, CINAHL with full text, and Health Sources), Cochrane Library, Scopus, Web of Science, Science Direct, WorldCat, and Google Scholar from the 18th to 24th of June 2020 and updated in November 2021 for peer review papers in English. Study design limitations were removed. The search strategy was piloted to check the appropriateness of keywords and databases. The results were reviewed by the research team to ensure the validity of the search strategy in PubMed. A hand search was conducted of the references of the included studies and the World Health Organization (WHO) website. Each search was adequately documented as illustrated in supplementary file 1. The Peer Review of Electronic Search Strategies (PRESS) statement guided this study’s electronic search strategy (McGowan et al. 2016). All citations were managed using the EndNote X9 reference manager.

### Selection of articles and eligibility criteria

The principal investigator conducted the database searches and title screening using this study’s eligibility criteria. The search strategy and screening tools were piloted to calibrate operators and increase consistency and fine-tune the methods. A second reviewer reviewed the retrieved titles to ensure completeness before the abstract screening. Subsequently, the cleaned EndNote library was shared among the review team after the removal of duplicate titles. Using an electronic screening tool developed in Google forms, two reviewers independently screened the abstracts and full texts and categorized them into “include” or “exclude” categories based on this study’s eligibility criteria. The review team met throughout the screening process and resolved the discrepancies between the two reviewers at the abstract screening stage through discussions until a consensus was reached even though there were no significant disagreements among the reviewers. It was decided that the articles will be selected on a minimum agreement of at least 50% between the two reviewers due to the complex and specialized field the review would entail. The second reviewer, however, resolved the discrepancies between the principal investigator and the third reviewer at the full-text screening phase. The PRISMA flow diagram was used to account for all the articles. This study’s eligibility criteria used are outlined below:

#### Inclusion criteria

- Studies that involved unnatural deaths
- Studies that focused on Forensic Medicine (autopsies/post-mortem)
- Articles that reported data practices such as use, benefits, and challenges
- Published from 2008 to 2021
- Published in the English language
- All study designs

#### Exclusion criteria

- Articles that do not involve Forensic Medicine/Pathology and/or autopsies
- Studies with no clear targeted population
- Studies where full-text articles could not be obtained
- Articles reporting photo capture/imaging programs
- Non-English publications

### Charting the data

The data for this study were collected using a spreadsheet comprising of the following: bibliographic details, publication year, study design, study setting, data practices relating to Forensic Medicine, uses, benefits, challenges, and conclusion and recommendations. The form was pilot tested by two reviewers independently, and all discrepancies were resolved before its usage. Finally, two reviewers performed the data extraction using both inductive and deductive approaches. Subsequent discrepancies were resolved through discussion by the review team.

### Collating, summarizing, and reporting the results

Thematic content analysis was conducted for this study. The emerging themes and subthemes relating to data practices in Forensic Medicine were collated, summarized, and reported narratively. However, the bibliographic details of the included studies such as design and publication year were reported quantitatively and presented as percentages.

## Results

**Fig. 1:**
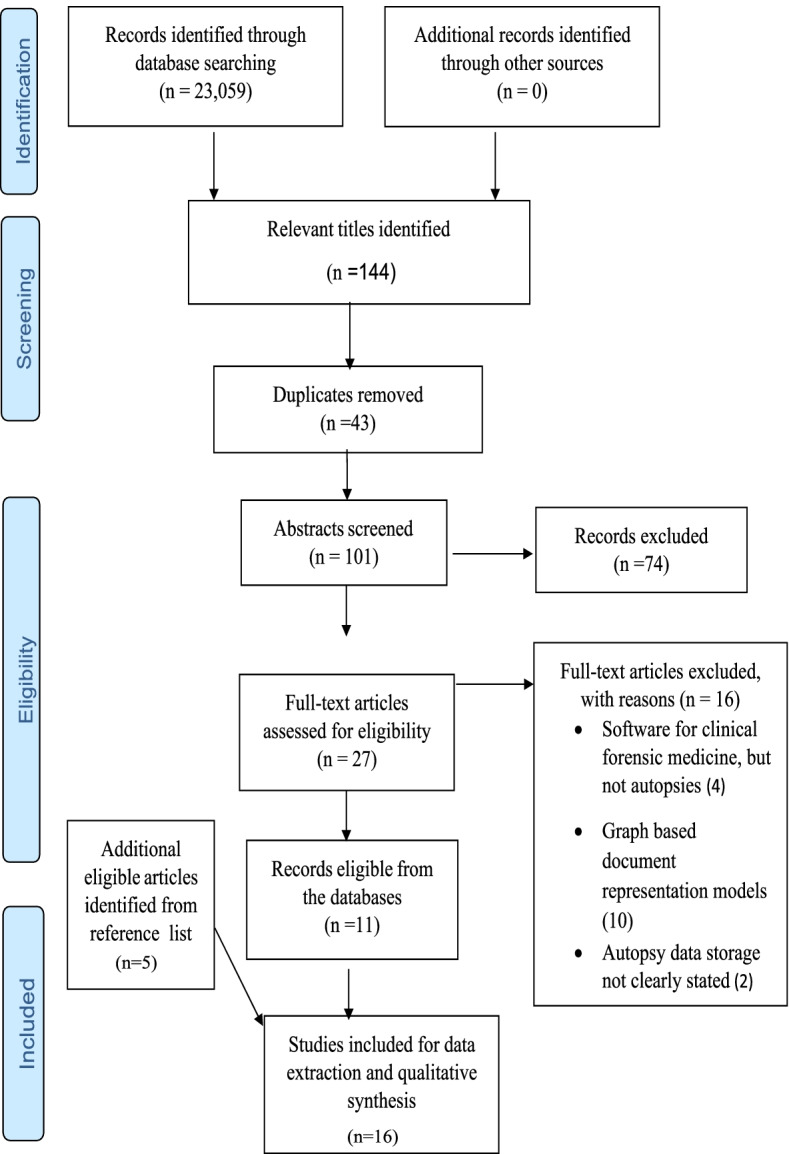
PRISMA 2009 flow diagram

Of the total of 23,059 search yields, 144 were deemed relevant from the title screening phase based on this study’s eligibility criteria. Of the of 144 titles, 43 duplicates identified were removed, and then, 74 and 16 articles were removed at the abstract and full-text screening phases, respectively. The 16 articles removed at the full-text screening phase focused on software for clinical forensic medicine, and graph-based document representation models and others did not report the data storage method. Therefore 15 articles were finally deemed eligible for inclusion for data extraction (Fig. 1). Following reviewer indication, an additional article was added to the results after the full-text screening for a total of 16 articles to be included in the study.

### Characteristics of included publications

Table 1provides a summary of the main characteristics of the articles included. The included articles were published between 2008 and 2019. Eight of the 16 articles were published between 2017 and 2019 (Soto Martinez et al. 2019; Dunstan 2019; Fowler et al. 2018; Hargrove et al. 2018; Dennis et al. 2018; Saar et al. 2017; Hofmeister et al. 2017; Ottaviani et al. 2017). Most of the studies were published in the USA (Soto Martinez et al. 2019; Fowler et al. 2018; Hargrove et al. 2018; Levy 2015; Blair et al. 2016)and Australia/New Zealand (Willcox et al. 2020; Dunstan 2019; Saar et al. 2017; Lyndal et al. 2016; Pearse 2012) and three from European countries. Two publications were from LMIC (Nigeria, Mexico), and the remaining were from high-income countries (Italy, Denmark, USA, Australia, and New Zealand, Japan, Switzerland, Canada (Fig.1). The article types included brief communication (Ottaviani et al. 2017; Kiuchi et al. 2013), original studies (Dunstan 2019; Saar et al. 2017; Hofmeister et al. 2017; Levy 2015; Aghayev et al. 2008), author manuscript (preprint) (Blair et al. 2016), retrospective descriptive studies (Soto Martinez et al. 2019; Fowler et al. 2018; Hargrove et al. 2018; Dennis et al. 2018; Kipsaina et al. 2015; Colville-Ebeling et al. 2014), a single systematic review (Lyndal et al. 2016), and an annual/government report (Canada 2012).

**Table 1:** General characteristics of included studies

Criterion	Number	Percentage
**Publication year**		
2008–2012	2	12.5%
2013–2016	6	37.5%%
2017–2019	8	50%
**Publication type**		
Brief communication	2	12.5%
Original article/review	5	31.25%
Retrospective descriptive	6	37.5%
Systematic review	1	6.25%
Annual/government report	1	6.25%
Author manuscript	1	6.25
**Sector/country**		
Nigeria	1	6.25%
Multiple countries including Mexico, Central America	1	6.25%
Tokyo/Japan	1	6.25%
Canada	1	6.25%
Australia/New Zealand	4	25%
Switzerland	1	6.25%
Denmark	1	6.25%
USA	5	31.25%
Italy	1	6.25%
**Data system coverage**		
National	6	37.5%
Almost complete national coverage	4	27%
Provincial/states	5	31.25%
Other/review	1	6.25%
**Uses**		
Diagnostic models/education	3	18.75%
Research	15	93.75%
Statistics	6	37.5%
Inform policy	5	31.25%
Information sharing	5	31.25%
**Benefits**		
Wide coverage	9	56.25%
Contribution to policies and epidemiology	10	62.5%
Data standardization	4	27%
Information security	2	13%
Anonymisation of data	3	20%
Data quality control	7	47%
Information sharing	6	37.5%
**Challenges/limitations**		
Underreporting/low-case numbers	10	67%
Data quality/validity (missing cases)	8	53%
Poor information exchange systems	8	53%
No standardization of data	5	33%
Ethical considerations	2	13%
Reporting bias/error/selection bias	4	27%
**Implications of results**		
Beneficial for research, policy making, prevention strategies, information exchange	16	100%
Level of sophistication: Simple beneficial database	1	7%
Sophisticated database: Multiple stakeholder involvement therefore beneficial outside Forensic Medicine-private companies, etc	5	31.25%

### Findings

Table 2 present a summary of these findings. A brief narrative of the finding is reported under the following main themes: data management and practice systems, benefits/uses of the data management and practice systems, and challenges/limitations of the data management and practice systems.

**Table 2:** Summary of study findings

Author, year	Country	Data reporting systems	Benefits/uses of reporting system	Challenges/limitations of reporting system
Aghayev, 2008	Switzerland	Virtopsy-repository of autopsy and radiological data	Digital and standardized documentation tool for forensic-radiological and pathological findings and comparison, epidemiological tool, archiving and distribution of data, continuing research and education, tracking tool for quality control, telemedicine, anonymous data passes to the central server	Tedious-require a large amount of time to enter data, language limited
Canada, 2012	Canada	Canadian Coroner and Medical Examiner Database National Coronial Database	Centralized source of data, closed cases, thorough analysis of data quality and integrity, standardization of cases, enhance information exchange for policy making	Under coverage (minimal), not completely reflective (not natural cases), may not link toxicology
Kiuchi, 2013	Japan	Institutional Database System located in each institute and containing personal information, and the Central Anonymous Database System located in the University Hospital Medical Information Network	Pathologists can retain, check or search personal databases, use by authorized users, proofreading system or quality and comprehensiveness of data, anonymization of data, hard copy of death certificate, real-time preservation, storage	Cases may be identified even after anonymization, informed consent, or ethical approval not required for data submission or analysis
Colville-Ebeling, 2014	Denmark	Database with authorized access only belonging to Department of Forensic Medicine	Security confidentiality maintained with authorized confidential (social security numbers used) access only, detailed information including autopsy reports, police reports, crime scene reports, and almost complete coverage of region, can be cross-referenced	No current validity methods, some information varies in a case-to-case basis
Kipsaina, 2015	Nigeria	Fatal injury surveillance system	First known fatal injury surveillance system in Nigeria to formulate injury prevention policies, created using available resources, standard data collection form by one data collector	Underreporting due to social circumstances, cultural influences, etc. Collection of data during a short period
Levy, 2015	USA	Varied systems: Medical Examiners and Coroners Alert System, ME/Coroner Information Sharing System, NVDRS, National Missing and Unidentified Persons System	MECAP-9000 product recalls or standard development; MECISP-tried to standardize data; NVDRS-improved research into preventable deaths; NamUs-resolved 9000 cases of missing or unidentified persons	Voluntary entry of information; manual or semi-automated; limited resources, no standardization of data collection, no communication or information sharing methods
Lyndal, 2016	Australia and New Zealand	National Coronial Database	Comprehensive coverage of cases within depth detail, reliable with high-quality data due to consistency with other data sources, a useful tool in death investigation and research on public health and safety-helped identify trends in specific death types-valuable to researchers and injury prevention practitioners/policy makers, identify hazards, inform the development of prevention strategies, assess their effectiveness, accurate estimation of mortality	Unavailability of data due to open cases, missing information, coding errors-underreporting of relevant cases, incomplete datasets, misclassification, inability to detect trends-erroneous reporting of decreasing trends due to small number of cases, selection bias or reporting bias (due to interest in closing the certain high-profile case)
Ottaviani, 2017	Italy	Creation of a web portal for a national data bank registry	Enhance epidemiological correlations with risk factors to provide further insight into SIDS	Requires consent from family
Hofmeister, 2017	Multiple countries including Mexico and Central America	Standard reporting form, a software application AM/PM Database, single or multi-user, in two languages	Assists in the identification of missing persons in armed conflicts and migration, one consolidated centralized system	Co-ordination of data exchange is difficult, needs training and technical staff because multiple countries involved, infrastructure and funding required, difficulty standardization of data
Saar, 2017	Australia and New Zealand	Internet database maintained by an IT support team nightly or weekly uploading of data	Standardization of data, more than 100 ethically approved research or monitoring projects as an ongoing data source, 215 publications, informed manufacturing changes, regulatory changes, awareness campaigns, suicide prevention initiatives, and coronial recommendations	Funding required for maintenance and support of multiple agencies (Allocation of court resources)
Hargrove, 2018	USA	Data is manually entered or imported into Epi Info V.7 with five data entries: death certificate data, coroner report data, autopsy report data, toxicology report data, and prescription drug report data	Enhanced surveillance data, data quality improvement, intervention and policy implementation, multi-stakeholder involvement	Underreporting of cases by hospital physicians was identified
Fowler, 2018	USA	State-based surveillance system. Data collected from individual information sources are entered into the NVDRS online data entry system with quality checks, training, and quality checks	Used to define public health priorities, develop and evaluate programs and policies, conduct research. Online platform simplified system operations and management, improved timeliness of data entry and reporting, enhanced flexibility	Not nationally representative, availability completeness and timeliness of data dependent on partnerships among state health departments, sharing and communication challenges, incomplete data, toxicology data not consistently collected, different classifications of deaths, different coding, protective factors not collected
Dennis, 2018	Australia	National Coronial Database funded by a governmental association	Study showed an opportunity to institute preventative measures (CPR training and defibrillators), outcomes improved possibly due to an increase in witnessed events, looked at preparticipation screening	Low number of cases, retrospective, not standardized data, missed cases
Soto Martinez, 2019	USA	A web-based platform, the Research Electronic Data Capture (REDCap) platform. Migration of the IID to a REDCap platform provided an opportunity to redesign the database to capture internal, external, and skeletal injuries with greater detail	Used to develop statistically sound diagnostic models, reliable, autopsy gold standard	Small number of cases, errors, complexity of observations, autopsy sample therefore not complete complement of injuries
Dunstan, 2019	Australia and New Zealand	National Coronial Information System	Data sharing with statistical and research data, identify mortality trends, formulate effective recommendations in the prevention of death and injury, contribution to health policy and prevention, access levels, ethics application process to obtain data	Reports only on closed cases, although nationally standardized information availability may vary, not all fatalities reported, no transcripts, photographic evidence or witness statements, non-fatal injury data, or information on the perpetrator
Blair, 2016	USA	NVDRS created in response to a 1999 Institute of Medicine report outlining the need for a national fatal intentional injury system, the first multistate system to provide detailed information on circumstances precipitating violent deaths, the first to link multiple source documents on violence-related deaths to enable researchers to understand each death more completely, and the first to link multiple deaths that are related to one another (e.g., multiple homicides, multiple suicides, and cases of homicide followed by the suicide of the suspected perpetrator)	Detailed circumstantial information regarding homicides and suicides enables research to be conducted to provide evidence basis for prevention programs. State health departments utilize information from the systems to identify areas of need, evaluate state policies and areas requiring intervention to produce targeted solutions or interventions. Improved elder abuse and neglect surveillance and targeted intervention programs	As of the publication of the article, the system was in place in 32 states and not national for complete surveillance

### Types of data management and practice systems

All the reviewed studies described the use of electronic-based systems which ranged in complexity from a simplified Excel spreadsheet (Kipsaina et al. 2015)to a more complex system based on the use of a web portal (Kiuchi et al. 2013)and use at the state level and national levels, the National Violent Death Reporting System ((Blair et al. 2016), and the National Coronial Information System systems (Dunstan 2019; Saar et al. 2017; Lyndal et al. 2016). The national system received full technical support. In some cases, such as mentioned in the Nigerian study, data was collected by a single user and was based on tools developed by WHO-Monash University fatal injury surveillance manual (2012). It was found to be acceptable and timely with good data quality which was representative and specific due to a single experienced user. Other systems sourced data from multiple users and departments such as the Forensic Medicine Department, Police, Psychologist. Many of the systems were consolidated centralized systems that were used in the Forensic Medicine Departments (*n*= 10; 62.5%) (Dunstan 2019; Saar et al. 2017; Hofmeister et al. 2017; Ottaviani et al. 2017; Blair et al. 2016; Lyndal et al. 2016; Pearse 2012; Aghayev et al. 2008; Canada 2012). Access to all the systems was strictly controlled either through single/multiple user authorization (Dunstan 2019; Hofmeister et al. 2017; Lyndal et al. 2016; Kiuchi et al. 2013; Aghayev et al. 2008; Colville-Ebeling et al. 2014), or data are captured in a spreadsheet created in Excel (Kipsaina et al. 2015). The articles based on systems that reported the best utility were those that allowed greater flexibility and lend themselves to state-based use (Fowler et al. 2018; Blair et al. 2016). In these cases, the systems received government funding and technical support (Hofmeister et al. 2017; Blair et al. 2016; Lyndal et al. 2016; Aghayev et al. 2008; Colville-Ebeling et al. 2014; Canada 2012).

### Benefits/uses of the reported data management and practice systems

In 9 (56%) of the articles, the wide coverage of cases increased the value of the system for those who used the data (Dunstan 2019; Fowler et al. 2018; Hargrove et al. 2018; Saar et al. 2017; Hofmeister et al. 2017; Ottaviani et al. 2017; Blair et al. 2016; Colville-Ebeling et al. 2014; Canada 2012). The benefits reported included the use to formulate injury prevention policies and enhance epidemiological studies (*n*= 10; 62.5%) (Dunstan 2019; Fowler et al. 2018; Hargrove et al. 2018; Saar et al. 2017; Ottaviani et al. 2017; Levy 2015; Blair et al. 2016; Lyndal et al. 2016; Colville-Ebeling et al. 2014; Canada 2012). Many of the cited systems contributed and supported research (*n*= 10; 62.5%) (Arksey and O'Malley 2005; Dunstan 2019; Fowler et al. 2018; Saar et al. 2017; Ottaviani et al. 2017; Levy 2015; Blair et al. 2016; Lyndal et al. 2016; Colville-Ebeling et al. 2014; Canada 2012). Although only a few systems had standardization of data as an attribute (*n*= 4; 27%) (Fowler et al. 2018; Hofmeister et al. 2017; Aghayev et al. 2008; Kipsaina et al. 2015), two reported the benefits of information security (*n*= 2; 13%) (Kiuchi et al. 2013; Colville-Ebeling et al. 2014)and 3 (20%) included the anonymization of data as a characteristic (Kiuchi et al. 2013; Aghayev et al. 2008; Colville-Ebeling et al. 2014). Some of the systems mentioned the use of their own data quality control measures (*n*= 7; 47%) (Dunstan 2019; Hargrove et al. 2018; Saar et al. 2017; Blair et al. 2016; Lyndal et al. 2016; Kiuchi et al. 2013; Canada 2012). Only six (37.5%) of the reported systems allowed for ease of data sharing/information exchange for the research and policy implementation (Dunstan 2019; Saar et al. 2017; Blair et al. 2016; Lyndal et al. 2016; Aghayev et al. 2008).

The *data management and practice systems* serve as repositories for information to inform about international best practices and supported the development of diagnostic models (Soto Martinez et al. 2019; Levy 2015; Fowler et al. 2018). The systems were also seen as a conduit for information sharing once privacy challenges were tackled (Levy 2015; Colville-Ebeling et al. 2014; Aghayev et al. 2008; du Toit-Prinsloo and Saayman 2012). The provision of data for research was a large contribution to most of the databases establishment (Soto Martinez et al. 2019; du Toit-Prinsloo and Saayman 2012; Dennis et al. 2018; Dunstan 2019; Fowler et al. 2018; Lyndal et al. 2016; Saar et al. 2017; Levy 2015; Pearse 2012; Kiuchi et al. 2013; Aghayev et al. 2008; Kipsaina et al. 2015; Colville-Ebeling et al. 2014). The systems contributed to the institution of preventative measures by identifying risk factors and by predicting the possible outcomes by using the data available in the reporting systems (du Toit-Prinsloo and Saayman 2012; Fowler et al. 2018; Blair et al. 2016; Lyndal et al. 2016). Improvement of surveillance and statistics related to epidemiology was notable in some studies (Saar et al. 2017; Ottaviani et al. 2017; Blair et al. 2016), and this further informed policy changes, contributed to the institution of new policies, and the reformation of laws (Bagher et al. 2015; Lyndal et al. 2016; Saar et al. 2017; Blair et al. 2016; Pearse 2012).

### Challenges/limitations of the data management and practice systems

In one of the systems included for review, the authors described that data were captured voluntarily. This however led to inconsistencies, underreporting and time lag that impacted the usability of the data for research and epidemiology (PRISMA 2018; Dunstan 2019; Fowler et al. 2018; Hofmeister et al. 2017; Kipsaina et al. 2015). Some of the other challenges reported (*n*= 8; 53%) in service work and research included underreporting, low-case numbers, or use of closed cases only which generally impacted on epidemiology and research (Soto Martinez et al. 2019; Hargrove et al. 2018; Dennis et al. 2018; Saar et al. 2017; Blair et al. 2016; Lyndal et al. 2016; Kipsaina et al. 2015; Canada 2012). In the articles that cited data quality and validity challenges (*n*= 8; 53%) missing information was also reported as a limitation (Dunstan 2019; Fowler et al. 2018; Saar et al. 2017; Levy 2015; Blair et al. 2016; Lyndal et al. 2016; Colville-Ebeling et al. 2014). Data exchange and entry limitations were also reported as an issue (*n*= 8; 53%) (Dunstan 2019; Fowler et al. 2018; Saar et al. 2017; Hofmeister et al. 2017; Levy 2015; Lyndal et al. 2016; Aghayev et al. 2008), and these barriers may be in part be due to an inability to institute standardization practices for data capturing (*n*= 5; 33%) (Fowler et al. 2018; Dennis et al. 2018; Hofmeister et al. 2017; Levy 2015; Blair et al. 2016). Ethical considerations such as consent for autopsy or submitting data for analysis and research also impacted the use of the systems (*n*= 2; 13%) (Ottaviani et al. 2017; Kiuchi et al. 2013). Factors such as case-reporting bias, reporting errors, and bias in case selection also impacted case numbers and research (*n*= 4; 27%) (Soto Martinez et al. 2019; Blair et al. 2016; Lyndal et al. 2016; Canada 2012).

## Discussion

**Fig. 2:**
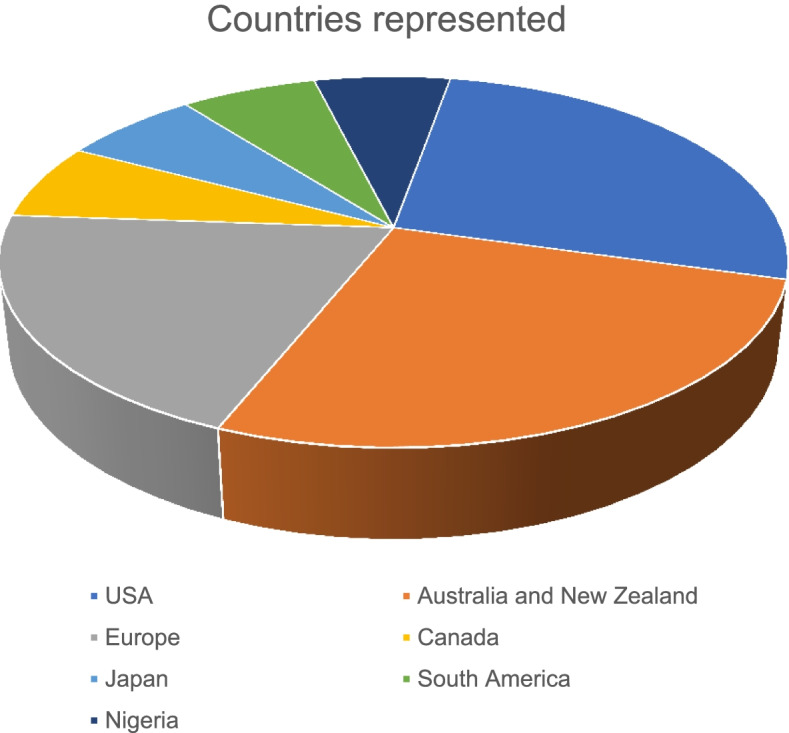
Countries where publications originated

This study sought to map the evidence on data management and practice systems in Forensic Medicine. The results show that several data management and practice systems exist. However, most of the existing systems were from high-income countries with few in LMICs based on this study’s eligibility criteria. This review was informed by the need to collect systematic evidence on the data management and practice systems being used in forensic medicine and the possible lessons and applicability to LMIC contexts where paper reporting is still being used. The lack of information on data management and practice systems is visible in that only two of the articles included for review reported on studies from the LMIC context. Half the publications (50%) were published during and after 2017. This trend may be due to the greater access, use, and reporting of electronic data reporting systems. The inclusion of Forensic Pathology reports on databases also captures information that is not routinely captured in vital registration statistics. The existing data management systems in forensic medicine reported in the included articles have several benefits. Nonetheless, the included articles also reported several challenges about those existing data management systems (see Fig. 2).

To the best of our knowledge, this scoping review is the first present evidence on data management and practice systems in Forensic Medicine. Therefore, we cannot compare our findings. Nonetheless, the literature shows that electronic data reporting systems are relevant and were developed from the recognition of coronial data as not only a part of the death investigation but as a contributor to preventable death research and public health initiatives. For instance, in an article by Bruce Levy which discussed the United States of America systems currently in place to support Forensic Pathology and death investigation, the CDC implemented the National Violent death Reporting System in response to a report describing the need for a national fatal intentional injury surveillance system. Initially, the system started in six states but later was expanded to 18 states as stated in the publication (Fowler et al. 2018). According to the CDC website, and an article published in 2019 regarding its future directions, the program has now expanded to all 50 states and is constantly being updated and improved for data sharing (Blair et al. 2016; CDC, 2012). Other systems developed in the USA included a state-wide comprehensive multisource drug overdose fatality surveillance system in Kentucky (developed in response to drug overdoses cited as a public health crisis) and a database related to infant and child abuse (Soto Martinez et al. 2019; Hargrove et al. 2018). The burden of sudden infant death syndrome and the large number of cases that remain unexplained led to the passing of legislation in Italy (2006) that fetuses and infants, from 25 weeks of gestation to one postnatal year, who died suddenly and unexpectantly should be sent to the University of Milan, Italy, for a postmortem with parental consent (Ottaviani et al. 2017). An Italian research center developed a web portal for a national bank registry which has been set up to centralize records retrieved from regions across Italy which hopes to contribute data for epidemiology and study into risk factors for sudden unexpected death in infants. Canada instituted a National Coroner and Medical Examiner database to detect emerging trends and hazards for the prevention of avoidable deaths (Canada 2012). Tokyo is recognized as a technology hub, and the latest inclusion of a Legal Medicine Information System for forensic systems is discussed in an included article (Kiuchi et al. 2013). Using the information system, Forensic Pathologists and other staff can register and search for institutional autopsy information, print death certificates, and extract data for research and analysis. Switzerland created a tool called the Virtopsy-a centralized database in Forensic Medicine for analysis and comparison of radiological and autopsy findings (Aghayev et al. 2008). It is a database currently created but not in routine use as it has not been validated. The database compares autopsy and radiological data with photograph storage. In the Nigerian study, recognition of the poorly representative mortality injury surveillance system prompted the authors to institute an electronic injury surveillance system (Kipsaina et al. 2015). The system included features based on a South African initiative namely the National Injury Mortality Surveillance System (NIMSS) which due to logistical reasons and lack of funding was deemed unfeasible and discontinued (Prinsloo 2019). Articles related to NIMSS did not meet the inclusion criteria of the current study. Although the tools that were utilized were already existing making the system feasible and sustainable, but an appropriate infrastructure needs to be in place to maintain the system (Prinsloo 2019).

The recognition and understanding of violent deaths require the collection of accurate, timely, and comprehensive surveillance data to implement preventative measures (du Toit-Prinsloo and Saayman 2012; Prinsloo 2019; Saar et al. 2017; Blair et al. 2016; Pearse 2012). The wealth of information collected by Forensic Pathologists can be effectively used in public health and safety initiatives, policies, and legislation (Dunstan 2019; Fowler et al. 2018; Blair et al. 2016). The databases have been credited as an evidence base for awareness-raising and death prevention initiatives informing research, policy development, and coronial investigation (Dunstan 2019; Blair et al. 2016). It encourages information exchange, standardization, and implementation of investigation protocols, and research. It has contributed to the publication of more than a hundred articles in a broad range of journals (Blair et al. 2016; Lyndal et al. 2016). Access, tracking, and centralization of data can result in the improvement of scientific and investigative processes with the implementation of international standards and best practices (Fowler et al. 2018; Saar et al. 2017; Blair et al. 2016; Kiuchi et al. 2013). The implementation of the system improved the quality of the surveillance data and the standardization of data. Furthermore, using and linking multiple sources of data-enabled valuable information to be extracted and translated for the identification of vulnerable populations at risk and provided evidence to implement a new legislature (Fowler et al. 2018; Levy 2015). It can be cost-effective and impact public health to reduce waste of resources and improve public initiatives. This data can further be used to develop diagnostic models to better inform clinical decision-making (Soto Martinez et al. 2019). It was recognized that the tool used must be comprehensive and adaptable as data management systems are indispensable as part of forensic investigations (Hofmeister et al. 2017; Levy 2015). It can be remodeled to an online platform that simplified system operations and management, improved timeliness of reporting, and increased adaptability which creates an opportunity for expansion to multiple sites.

There were several limitations discussed which primarily involved feasibility of the system, accuracy, availability, and completeness of data, involvement of relevant stakeholders, and the absence of morbidity data (Lyndal et al. 2016). Collaboration may address challenges of sharing and merging and analyzing of data. Developing policies regarding storage, quality review, and access of the data for analysis may address privacy challenges. Important issues discussed included the protection of data privacy which can be overcome by anonymization of data on the central server and the case sensitive information can be stored on the local server (Kiuchi et al. 2013; Aghayev et al. 2008). The use of the Internet can be a cost-effective solution whereas more sophisticated databases require time and resources and a necessary framework that involves policies, staff, training, quality control, and support. The issues relating to the systems are the limited resources for death investigations (both human factors and technology). The involvement of various stakeholders to support data-sharing programs in Forensic Pathology can relieve the financial strain (Dunstan 2019; Fowler et al. 2018; Hargrove et al. 2018; Dennis et al. 2018; Saar et al. 2017; Hofmeister et al. 2017; Ottaviani et al. 2017; Levy 2015). The Forensic Medicine Departments can utilize simple and available tools that can advance standardization of data collection, storage, and reporting because of the central role they play in reporting provincial/national data (Kipsaina et al. 2015). The reviewed articles included a great variety of systems that could lend themselves to use in LMIC contexts. Apart from its limitations, the autopsy/coronial data reporting systems are recognized as an essential tool for monitoring the prevalence and incidence of violence related to fatal injuries.

### Strengths and limitations

This study is the first scoping review that systematically mapped literature relating to data practices in Forensic Medicine globally. A major strength of our study method is that it permits the inclusion of all study designs and the development of a protocol that ensures reproducibility. Moreover, we conducted a thorough search using a comprehensive search strategy which enabled us to capture the most relevant articles to answer the review question. However, the articles selected were limited to Forensic Medicine, keywords, and its data collection methods of autopsy records; therefore, articles that entail electronic methods for the data collection in other medical departments were excluded. The limitation was due to the focus of the study being on the value of these systems to preventative programs rather than treatment. The keywords to be used in the search strategy are broad and may not identify specialized studies in data management. Only articles in English will be used. Nonetheless, the finding produced by this study is useful to inform further research, particularly LMICs.

### Recommendations

The general consensus from the articles is that a data management and practice system containing coronial/medical examiner/Forensic Pathology data is beneficial for research, policymaking, prevention strategies, and information exchange for education. Due to resource limitations in some low- to middle-income countries, the database can be done by using available resources to create a limited database (Kipsaina et al. 2015). Although if multiple stakeholders can be involved to formulate and fund a nationally representative information system, this can be wholly beneficial, not only to the community and government but may also impact on private companies with regard to product development and reformation due to its comprehensive coverage of preventable deaths (Dunstan 2019; Hargrove et al. 2018; Saar et al. 2017; Blair et al. 2016; Lyndal et al. 2016).

## Conclusions

This scoping review summarized the evidence on data management and practice systems and their benefits and challenges in forensic medicine. The very appropriate use of words in an article’s title “Saving Lives Through the Power of Data” reiterates the appropriate use of information from preventable deaths. The imperative to use autopsy data for statistically relevant but also representative data can be time-consuming and an arduous task. The electronic systems, ranging from the most sophisticated (NCIS, NVDRS, Virtopsy Switzerland, Tokyo CDISC/ODM) to those created considering resource limitations (Nigerian trial), are cited as beneficial to the pathologist, researchers and for public health. The limitations to implementing electronic systems may include the reluctance of various stakeholders (such as government agencies) to participate and the need for additional funding to sustain more sophisticated database systems. However, the use of simple and available tools such as in the Nigerian trial still managed to contribute to statistically relevant data for impactful research.

## Supplementary Information

**Additional file 1.**

## Data Availability

All data generated or analyzed during this study will be included in the published scoping review article.

## Abbreviations

CADS: Central Anonymous Database System and IDS Institutional Database System (Part of CDISC/ODM Clinical Data Interchange Standards Consortium Operational Data Model)
CDC: Centres for Disease Control and Prevention
ICRC AM/PM Database: International Committee of the Red Cross Antemortem/Postmortem Database
LMIC: Low- to middle-income countries
IID: Infant Injury Database
MeSH: Medical Subject Headings
NCIS: National Coronial Information System
NIMSS: The National Injury Mortality Surveillance System
NVDRS: National Violent death Reporting System
WHO: World Health Organization
