# Supplementary material for: Introductory evidence on data management and practice systems of forensic autopsies in sudden and unnatural deaths: a scoping review
Source: Egypt J Forensic Sci. 2022 Sep 19;12(1):38. doi: 10.1186/s41935-022-00293-3 (PMC9484346; doi:10.1186/s41935-022-00293-3)
Supplement: Supplementary file 1 — Additional file 1. [file 41935_2022_293_MOESM1_ESM.docx]

Supplementary material: Figures and tables


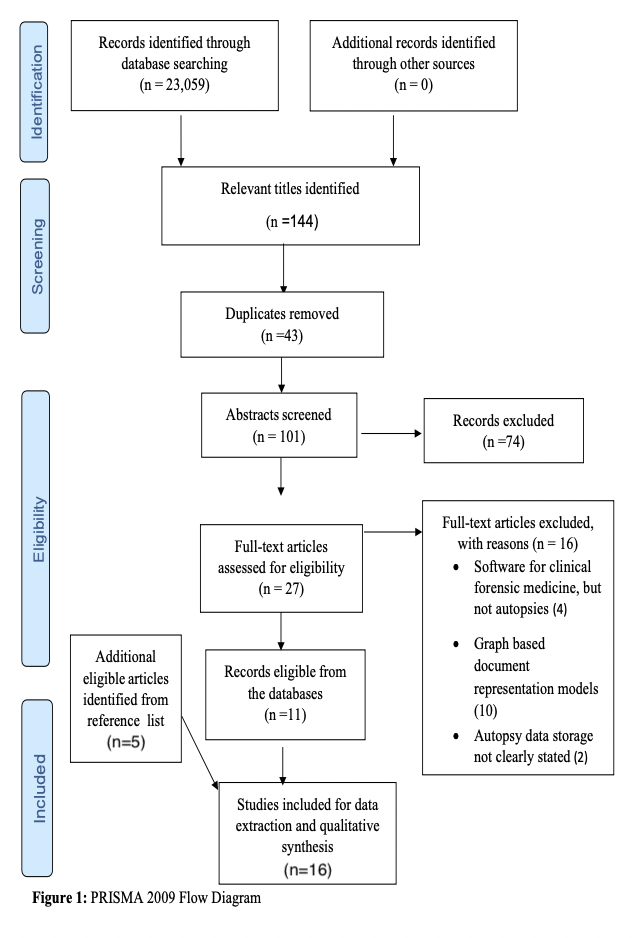


**Table 1:** General characteristics of included studies

| **Criterion** | **Number** | **Percentage** |
| --- | --- | --- |
| **Publication year**  2008-2012  2013-2016  2017-2019 | 2  5  9 | 13%  33%  56% |
| **Publication type**  Brief communication  Original article/review  Retrospective descriptive  Systematic review  Annual/government report  Author manuscript | 2  5  6  1  1  1 | 12.5%  31.25%  37.5%  6.25%  6.25%  6.25 |
| **Sector/country**  Nigeria  Multiple countries including Mexico, Central America  Tokyo/Japan  Canada  Australia/New Zealand  Switzerland  Denmark  USA  Italy | 1  1  1  1  4  1  1  5  1 | 6.25%  6.25%  6.25%  6.25%  25%  6.25%  6.25%  31.25%  6.25% |
| **Data system coverage**  National  Almost complete national coverage  Provincial/states  Other/review | 6  4  5  1 | 37.5%  27%  31.25%  6.25% |
| **Uses**  Diagnostic models/Education  Research  Statistics  Inform policy  Information sharing | 3  15  6  5  5 | 18.75%  93.75%  37.5%  31.25%  31.25% |
| **Benefits**  Wide coverage  Contribution to policies & epidemiology  Data standardisation  Information security  Anonymisation of data  Data quality control  Information sharing | 9  10  4  2  3  7  6 | 56.25%  62.5%  27%  13%  20%  47%  37.5% |
| **Challenges/limitations**  Underreporting/low case numbers  Data quality/validity (missing cases)  Poor information exchange systems  No standardisation of data  Ethical considerations  Reporting bias/error/selection bias | 10  8  8  5  2  4 | 67%  53%  53%  33%  13%  27% |
| **Implications of results**  Beneficial for research, policy making, prevention strategies, information exchange  Level of sophistication can range from simple  Multiple stakeholder involvement beneficial outside Forensic-private companies etc. | 16  1  5 | 100%  7%  31.25% |

**Table 2:** Summary of study findings

| **Author, year** | **Country** | **Data reporting systems** | **Benefits/uses of reporting system** | **Challenges/limitations of reporting system** |
| --- | --- | --- | --- | --- |
| {Aghayev, 2008 #15} | Switzerland | Virtopsy-repository of autopsy and radiological data | Digital and standardised documentation tool for forensic-radiological and pathological findings and comparison, epidemiological tool, archiving and distribution of data, continuing research and education, tracking tool for quality control, telemedicine, anonymous data passes to central server | Tedious-require large amount of time to enter data, language limited |
| {Canada, 2012 #14} | Canada | Canadian Coroner and Medical Examiner Database  National Coronial Database | Centralised source of data, closed cases, thorough analysis of data quality and integrity, standardisation of cases, enhance information exchange for policy making | Under coverage (minimal), not completely reflective (not natural cases), may not link toxicology |
| {Kiuchi, 2013 #32} | Japan | Institutional Database System located in each institute and containing personal information, and the Central Anonymous Database System located in the University Hospital Medical Information Network | Pathologists can retain, check or search personal databases, use by authorised users, proof reading system or quality and comprehensiveness of data, anonymisation of data, hard copy of death certificate, real time preservation, storage | Cases may be identified even after anonymisation, informed consent or ethical approval not required for data submission or analysis |
| {Colville-Ebeling, 2014 #31} | Denmark | Database with authorised access only belonging to Department of Forensic Medicine | Security-confidentiality maintained with authorised confidential (social security numbers used) access only, detailed information including autopsy reports, police reports, crime scene reports, almost complete coverage of region, can be cross referenced. | No current validity methods, some information varies in case-to-case basis |
| {Kipsaina, 2015 #28} | Nigeria | Fatal injury surveillance system | First known fatal injury surveillance system in Nigeria to formulate injury prevention policies, created using available resources, standard data collection form by one data collector | Under reporting due to social circumstances, cultural influences etc. Collection of data during short period. |
| {Levy, 2015 #22} | USA | Varied systems: Medical Examiners and Coroners Alert System, ME/Coroner Information Sharing System, NVDRS, National Missing and Unidentified Persons System | MECAP-9000 product recalls or standard development; MECISP-tried to standardise data; NVDRS-improved research into preventable deaths; NamUs-resolved 9000 cases of missing or unidentified persons | Voluntary entry of information; manual or semi-automated; limited resources, no standardisation of data collection, no communication or information sharing methods |
| {Lyndal, Bugeja, 2016 #25} | Australia and New Zealand | National Coronial Database | Comprehensive coverage of cases within depth detail, reliable with high quality data due to consistency with other data sources, useful tool in death investigation and research on public health and safety-helped identify trends in specific death types-valuable to researchers and injury prevention practitioners/policy makers, identify hazards, inform development of prevention strategies, assess their effectiveness, accurate estimation of mortality | Unavailability of data due to open cases, missing information, coding errors-underreporting of relevant cases, incomplete datasets, misclassification, inability to detect trends-erroneous reporting of decreasing trends due to small number of cases, selection bias or reporting bias (due to interest in closing certain high-profile case) |
| {Ottaviani, 2017 #30} | Italy | Creation of a web portal for a national data bank registry | Enhance epidemiological correlations with risk factors to provide further insight into SIDS | Requires consent from family |
| {Hofmeister, 2017 #27} | Multiple countries including Mexico and Central America | Standard reporting form, a software application AM/PM Database, single or multi user, in two language. | Assists in identification of missing persons in armed conflicts and migration, one consolidated centralized system | Co-ordination of data exchange difficult, needs training and technical staff because multiple countries involved, infrastructure and funding required, difficulty standardisation of data |
| {Saar, 2017 #27} | Australia and New Zealand | Internet database maintained by an IT support team nightly or weekly uploading of data | Standardisation of data, more than 100 ethically approved research or monitoring projects as an ongoing data source, 215 publications, informed manufacturing changes, regulatory changes, awareness campaigns, suicide prevention initiatives and coronial recommendations | Funding required for maintenance and support of multiple agencies (Allocation of court resources) |
| {Hargrove, 2018 #21} | USA | Data is manually entered or imported into Epi Info V.7 with five data entries: death certificate data, coroner report data, autopsy report data, toxicology report data, and prescription drug report data. | Enhanced surveillance data, data quality improvement, intervention and policy implementation, multi stake holder involvement | Under reporting of cases by hospital physicians was identified |
| {Fowler, 2018 #20} | USA | State based surveillance system. Data collected from individual information sources are entered into the NVDRS online data entry system with quality checks, training and quality checks. | Used to define public health priorities, develop and evaluate programs and policies, conduct research. Online platform simplified system operations and management, improved timeliness of data entry and reporting, enhanced flexibility | Not nationally representative, availability completeness and timeliness of data dependent on partnerships among state health departments, sharing and communication challenges, incomplete data, toxicology data not consistently collected, different classifications of deaths, different coding, protective factors not collected |
| {Dennis, 2018 #29} | Australia | Coronial database National Coronial Database funded by governmental association | Study showed an opportunity to institute preventative measures (CPR training and defibrillators), outcomes improved possibly due to increase in witnessed events, looked at preparticipation screening | Low number of cases, retrospective, not standardised data, missed cases |
| {Soto Martinez, 2019 #7} | USA | A web-based platform, the Research Electronic Data Capture (REDCap) platform. Migration of the IID to a REDCap platform provided an opportunity to redesign the database to capture internal, external, and skeletal injuries with greater detail. | Used to develop statistically sound diagnostic models, reliable, autopsy gold standard | Small number of cases, errors, complexity of observations, autopsy sample therefore not complete complement of injuries |
| {Dunstan, 2019 #23} | Australia and New Zealand | National Coronial Information System | Data sharing with statistical and research data, identify mortality trends, formulate effective recommendations in the prevention of death and injury, contribution to health policy and prevention, access levels, ethics application process to obtain data | Reports only on closed cases, although nationally standardised information availability may vary, not all fatalities reported, no transcripts, photographic evidence or witness statements, non-fatal injury data or information on perpetrator |
| {Blair, 2016 #15} | USA | NVDRS created in response to a 1999 Institute of Medicine report outlining the need for a national fatal intentional injury system, first multistate system to provide detailed information on circumstances precipitating violent deaths, the first to link multiple source documents on violence-related deaths to enable researchers to understand each death more completely, and the first to link multiple deaths that are related to one another (e.g., multiple homicides, multiple suicides, and cases of homicide followed by the suicide of the suspected perpetrator). | Detailed circumstantial information regarding homicides and suicides enables research to be conducted to provide evidence basis for prevention programs. State health departments utilise information from the systems to identify areas of need , evaluate state policies and areas requiring intervention to produce targeted solutions or interventions. Improved elder abuse and neglect surveillance and targeted intervention programs. | As of the publication of the article, the system was in place in 32 states and not national for complete surveillance |

Figure 2: Countries where publications originated
